# Autism Spectrum Disorder Studies Using fMRI Data and Machine Learning: A Review

**DOI:** 10.3389/fnins.2021.697870

**Published:** 2021-09-15

**Authors:** Meijie Liu, Baojuan Li, Dewen Hu

**Affiliations:** ^1^Engineering Training Center, Xi’an University of Science and Technology, Xi’an, China; ^2^College of Missile Engineering, Rocket Force University of Engineering, Xi’an, China; ^3^School of Biomedical Engineering, Air Force Medical University, Xi’an, China; ^4^College of Intelligence Science and Technology, National University of Defense Technology, Changsha, China

**Keywords:** autism spectrum disorder, functional connectivity, functional magnetic resonance imaging, machine learning, feature selection

## Abstract

Machine learning methods have been frequently applied in the field of cognitive neuroscience in the last decade. A great deal of attention has been attracted to introduce machine learning methods to study the autism spectrum disorder (ASD) in order to find out its neurophysiological underpinnings. In this paper, we presented a comprehensive review about the previous studies since 2011, which applied machine learning methods to analyze the functional magnetic resonance imaging (fMRI) data of autistic individuals and the typical controls (TCs). The all-round process was covered, including feature construction from raw fMRI data, feature selection methods, machine learning methods, factors for high classification accuracy, and critical conclusions. Applying different machine learning methods and fMRI data acquired from different sites, classification accuracies were obtained ranging from 48.3% up to 97%, and informative brain regions and networks were located. Through thorough analysis, high classification accuracies were found to usually occur in the studies which involved task-based fMRI data, single dataset for some selection principle, effective feature selection methods, or advanced machine learning methods. Advanced deep learning together with the multi-site Autism Brain Imaging Data Exchange (ABIDE) dataset became research trends especially in the recent 4 years. In the future, advanced feature selection and machine learning methods combined with multi-site dataset or easily operated task-based fMRI data may appear to have the potentiality to serve as a promising diagnostic tool for ASD.

## Introduction

As a pervasive neurodevelopmental disorder, autism spectrum disorder (ASD) is characterized by deficits in social communication and interaction and restricted and repetitive behaviors (Hull et al., 2017), which was known to be an urgent public health concern that could benefit from enhanced strategies to help identify ASD earlier (Jon et al., 2018). Diagnosed autism prevalence has risen dramatically over the last several decades (Nevison, 2014), and the causes have remained elusive, which have increasingly attracted numerous researchers to focus on it. Thus far, different advanced neuroimaging tools have been applied for ASD research, including structural and functional magnetic resonance imaging (MRI), positron emission tomography (PET), electroencephalography (EEG), magnetoencephalography (MEG), and novel protocols (Alessandro et al., 2017; Du et al., 2018). Among them, functional MRI (fMRI) studies involving task-based and resting-state fMRI (rs-fMRI) data occupy a large proportion. Especially with the appearance and development of freely available rs-fMRI databases, such as the Autism Brain Imaging Data Exchange (Martino et al., 2014)^1^, which provides functional and structural brain imaging datasets collected from more than 24 different independent sites, researchers from different countries have expanded a series of studies based on it. In this paper, the review about ASD classification is restricted to fMRI data for more specific analysis. The aforementioned fMRI data consist of rs-fMRI data and task-based fMRI data, which are collected from scanning the brain using fMRI technology while the subject is resting and performing a special task, respectively.

For the last decade, there have been a variety of methods proposed to investigate the potential difference between ASD patients and typical controls (TCs) from different levels using fMRI data. It is well known that machine learning methods have been widely applied to brain disorder research such as schizophrenia, depression, Alzheimer disease, ASD, and so on (Davatzikos et al., 2006; Fan et al., 2008; Cuingnet et al., 2011; Du et al., 2012; Arbabshirani et al., 2013; Zeng et al., 2014; Patel et al., 2015), especially with their rapid development. Recent progress in machine learning has been driven both by the development of new learning algorithms and theory and by the ongoing explosion in the availability of online data and low-cost computation (Jordan and Mitchell, 2015). Notwithstanding the fact that there have existed great quantity of research on classifications for ASD and the TCs, a specialized systematic review about them is lacking. While different approaches have different assumptions and advantages, a detailed review is important to help us understand the ways in which these approaches have been used. Wolfers et al. published a review about pattern recognition for neuroimage-based psychiatric diagnostics in which ASD was mentioned as a little part (Wolfers et al., 2015). Du et al. (2018) have reviewed relative literatures on classification and prediction of brain disorders using functional connectivity (FC) but not limited to ASD. Hyde et al. provide a comprehensive review of 45 papers utilizing supervised machine learning in ASD but not limited to fMRI data (Hyde et al., 2019).

**FIGURE 1:**
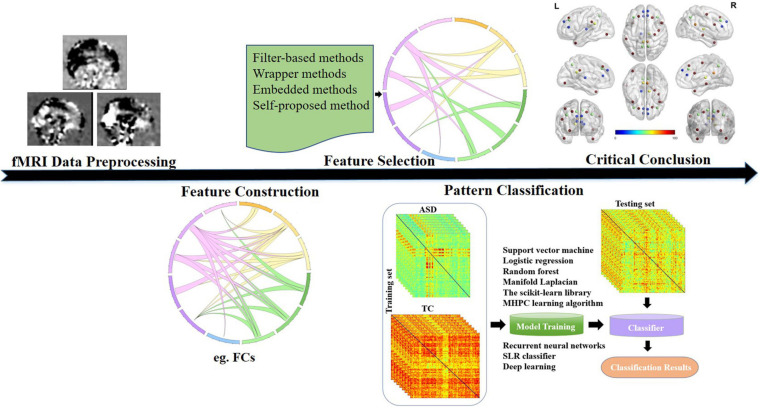
General process of ASD studies using fMRI data and machine learning (taking FC features for example). ASD, autism spectrum disorder; fMRI, functional magnetic resonance imaging; FC, functional connectivity.

In this paper, we exhibit a review by summarizing 47 literatures involving classifications using fMRI data between ASD patients and TCs. The general process of autism spectrum disorder studies using fMRI data and machine learning was illustrated in Figure 1. The purpose of this review is to (1) summarize relatively representative papers from the following aspects to find out their commonality and differentiation: classification features involved, machine learning methods, classification performance, and factors on classification results; (2) reveal critical consistent or novel conclusions about discriminant brain regions, networks, and explanations for behavioral characteristics of ASD in the literatures; and (3) give feasible work directions for future progress in the field.

## fMRI Data Sources

It is well known that spontaneous fluctuations in the blood oxygenation level-dependent (BOLD) signal, as measured by fMRI, present a valuable data resource for delineating the human neural functional architecture (Cole et al., 2010). fMRI, including rs-fMRI and task-based fMRI, has become one of the primary tools of cognitive neuroscience. From analysis of the papers detailed in Table 1, it was obviously found that there are mainly three types of fMRI data sources, which are self-acquisition data, the cooperation agency data, and freely available database. The self-acquisition data and the cooperation agency data are usually acquired from the same site with same acquisition parameters. Notably, all the task-based fMRI data involved belonged to the former two data sources. The well-known large freely available database is the ABIDE database. The data of the ABIDE database are collected from different sites in North America and Europe, which inevitably introduces heterogeneity into the dataset in terms of differences among fMRI scanners, data collection protocols, and participant populations (Ghiassian et al., 2016). The aggregation of data across multiple neuroimaging sites has become popular in recent years. Given that the sharing and combination of task-based fMRI data are significantly more challenging than rs-fMRI data (Poldrack and Gorgolewski, 2015), the large freely available database is mainly rs-fMRI data. Despite this, some efforts have been made to build an open repository for task-based fMRI data, such as the Open fMRI database (Poldrack et al., 2013; Poldrack and Gorgolewski, 2015). The advantage of the large database is that it contains more available data than the former two data sources and can satisfy more researchers’ purchase for large database analysis. Working toward the ultimate goal of deriving an automated diagnosis tool from the fMRI data classification, a large database is indispensable for further generalization. Unfortunately, the classification of the large database from different sites appears to be more challenging than that of the small database from the same site. In spite of the above challenge, the freely available database is getting increasing attention in the study of ASD. Notably, 33 of 47 literatures in Table 1 adopted the freely available database, which accounted for 70% of the total.

**TABLE 1:** Summary of ASD classification studies based on fMRI data.

Study	Participants	Data	Features	Feature selection	Machine learning method	Accuracy
Anderson et al. (2011)	40 ASD 40 TC	rs-fMRI data	Whole brain FCs between 7,266 ROIs	A two-tailed *t*-test for *p* < 0.001	Not detailed	79%
Wang et al. (2012)	29 ASD 29 TC	fMRI data with a cognitive control task	FCs between 106 ROIs (the AAL atlas)	None	Logistic regression	82.8%
Murdaugh et al. (2012)	13 ASD 14 TC	fMRI data with three stimuli experiments	Seed-based FCs FCs between 102 regions (the AAL atlas)	None	Logistic regression	96.3%
Nielsen et al. (2013)	447 ASD 517 TC	rs-fMRI data (the ABIDE dataset)	Whole brain FCs between 7,266 ROIs	None	Not detailed	60%
Uddin et al. (2013a)	20 ASD 20 TC	rs-fMRI data	Independent components	None	Logistic regression	83%
Deshpande et al. (2013)	15 ASD 15 TC	fMRI data with ToM task	FCs between 18 ROIs	Recursive cluster elimination	Linear SVM	95.9%
Just et al. (2014)	17 ASD 17 TC	fMRI data with a thinking task	Features obtained by factor analyses proposed by the author	The FA procedure	GNB	97%
Price et al. (2014)	30 ASD 30 TC	rs-fMRI data (the ABIDE dataset)	Dynamic FCs from multi-network	Self-proposed methods	Multi-kernel SVM	90%
Zhou et al. (2014)	127 ASD 153 TC	rs-fMRI data (the ABIDE dataset)	Integrated features	PCA and MRMR	SVM and Bayesian network	70%
Plitt et al. (2015)	59 ASD 59 TC	rs-fMRI data	FCs between ROIs from three atlases (the Destrieux atlas, the DiMartino atlas, and the Power atlas)	RFE	The scikit-learn library	76.67%(peak)
Dodero et al. (2015a)	42 ASD 37 TC	rs-fMRI data (the UCLA data)	FCs between 264 ROIs (the Power atlas)	None	Grass–Kernel based Manifold Laplacian	63.29%
Iidaka (2015)	312 ASD 328 TC	rs-fMRI data	FCs between 90 ROIs (the AAL atlas)	Threshold	Probabilistic neural network	89.4%
Chen et al. (2015)	126 ASD 126 TC	rs-fMRI data (the ABIDE dataset)	FCs between 220 functionally defined ROIs	PSO RFE	SVM Random forest	66% 90.8%
Chanel et al. (2016)	15 ASD 14 TC	fMRI data with emotional stimuli	The beta maps	RFE	SVM	92.3%
Ghiassian et al. (2016)	538 ASD 573 TC	rs-fMRI data (the ABIDE dataset)	Proposed HOG features	MRMR	MHPC learning algorithm	65%
Kassraian-Fard et al. (2016)	77 ASD 77 TC	rs-fMRI data (the ABIDE dataset)	FCs between 200 ROIs (the CC200 atlas)	None	SVM	63%
Odriozola et al. (2016)	23 ASD 22 TC	fMRI data with two visual oddball detection tasks	Multivariate activation patterns in the dorsal part of the anterior insula	None	SVM	85%
Abraham et al. (2017)	871 participants	rs-fMRI data (the ABIDE dataset)	FCs between ROIs from three atlases (the HO atlas, the Yeo atlas, and the CC200 atlas)	ICA and MSDL	The scikit-learn library	67% (peak)
Yahata et al. (2016)	74 ASD 107 TC	rs-fMRI data	FCs between 140 ROIs (the sulci-based anatomical atlas)	L1-SCCA	SLR classifier	85%
Dvornek et al. (2017)	539 ASD 573 TC	rs-fMRI data (the ABIDE dataset)	The resting-state fMRI time series	None	LSTM model^*a*^	68.5%
Rane et al. (2017)	539 ASD 573 TC	rs-fMRI data (the ABIDE dataset)	All voxels within the GM mask	None	the scikit-learn library	62%
Guo et al. (2017)	55 ASD 55 TC	rs-fMRI data (the ABIDE dataset)	FCs between 116 ROIs (the AAL atlas)	SAEs	DNN classifier^*a*^	86.36%
Bi et al. (2018)	45 ASD 39 TC	rs-fMRI data (the ABIDE dataset)	FCs between 90 ROIs (the AAL atlas)	random SVM cluster	RBF-SVM	96.15%
Heinsfeld et al. (2018)	505 ASD 530 TC	rs-fMRI data (the ABIDE dataset)	Whole brain FC between 7,266 ROIs	SAEs	DNN classifier^*a*^	70%
Aghdam et al. (2018)	116 ASD 69 TC	rs-fMRI and sMRI data (the ABIDE dataset)	Means of ROIs respectively for rs-fMRI, GM and WM (the AAL atlas)	None	DBN classifier^*a*^	65.56%
Zhao et al. (2018)	54 ASD 46 TC	rs-fMRI data (the ABIDE dataset)	Multi-level, high-order FCs	LASSO	multiple linear SVMs	81%
Soussia and Rekik (2018)	155 ASD 186 TC	rs-fMRI data (the ABIDE dataset)	High-Order Morphological Network	None	SIMLR based pairing + SVM	61.7%
Dekhil et al. (2018)	123 ASD 160 TC	rs-fMRI data	PSD	PSD with highest correlation with the 34 rs-fMRI atlases	RBF-SVM	91%
Bernas et al. (2018)	24 ASD 30 TC	rs-fMRI data	7 resting-state networks	Group-ICA	poly-SVM	86.7%
Bhaumik et al. (2018)	167 ASD 205 TC	rs-fMRI data (the ABIDE dataset)	FCs between Brodmann’s areas ROIs	Filter-based test and embedded Elastic Nets	Partial least square regression combined with SVM	70%
Kong et al. (2018)	78 ASD 104 TC	rs-fMRI data (the ABIDE dataset)	FCs between 148 ROIs (the Destrieux atlas)	F-score	DNN classifier^*a*^	90.39%
Li et al. (2018)	38 ASD 23 TC	rs-fMRI data (the ABIDE dataset)	FCs between 90 ROIs (the AAL atlas)	SSAE	DTL-NN classifier^*a*^	70.4%
Kazeminejad and Sotero (2019)	109 participants 342 participants 190 participants 137 participants 51 participants	rs-fMRI data (the ABIDE dataset)	FCs between 116 ROIs (the AAL atlas)	A sequential forward floating algorithm	Gaussian SVM	86% 69% 78% 80% 95%
Eslami et al. (2019)	505 ASD 530 TC	rs-fMRI data (the ABIDE dataset)	FCs between 200 ROIs (the CC200 atlas)	AE	A single layer perceptron^*a*^	80%
Fredo et al. (2019)	306 ASD 350 TC (400 participants for each sample)	rs-fMRI (the ABIDE dataset)	FCs between 237 ROIs (the Gordon’s cortical atlas the HO atlas)	Conditional random forest	Random forest	62.5% 65% 70% 73.75%
Niu et al. (2020)	408 ASD 401 TC	rs-fMRI data (the ABIDE dataset)	FCs between ROIs from three atlases separately (the AAL atlas, the HO atlas and the CC200 atlas)	None	The proposed multichannel DANN	73.2%
Liu Y. et al. (2020)	506 ASD 548 TC	rs-fMRI data (the ABIDE dataset)	FCs between 200 ROIs (the CC200 atlas)	Extra-tree	Linear-SVM	72.2%
Sherkatghanad et al. (2020)	505 ASD 530 TC	rs-fMRI data (the ABIDE dataset)	FCs between 392 ROIs (the CC400 atlas)	None	CNN classifier^*a*^	70.22%
Thomas et al. (2020)	620 ASD 542 TC	rs-fMRI data (the ABIDE dataset)	Nine summary measures	None	3D CNN classifier^*a*^	64%
Tang et al. (2020)	505 ASD 530 TC	rs-fMRI data (the ABIDE dataset)	FCs between 116 ROIs fMRI × ROI connectivity (the AAL atlas)	None	DNN classifier^*a*^	74%
Zhao et al. (2020)	45 ASD 47 TC	rs-fMRI data (the ABIDE dataset)	FCs, Lo-D-FCs and Ho-D-FCs between 116 ROIs (the AAL atlas)	A two-sample t-test and LASSO	Linear-SVM	83%
Huang et al. (2020)	505 ASD 530 TC	rs-fMRI data (the ABIDE dataset)	FCs between 200 ROIs (the CC200 atlas)	Graph-based feature-selection method	DBN classifier^*a*^	76.4%
Liu Y. et al. (2020)	403 ASD 468 TC	rs-fMRI data (the ABIDE dataset)	D-FCs between ROIs (the AAL atlas)	MTFS-EM	Multi-kernel SVM	76.8%
Kazeminejad and Sotero (2020)	493 ASD 530 TC	rs-fMRI data (the ABIDE dataset)	FCs between 200 ROIs (the CC200 atlas)	PCA	A multilayer perceptron^*a*^	64.4%
Yin et al. (2021)	403 ASD 468 TC	rs-fMRI data (the ABIDE dataset)	FC between 264 ROIs (the Power atlas)	An AE-based feature selection method	DNN classifier^*a*^	79.2%
Yang et al. (2021)	79 ASD 105 TC	rs-fMRI data (the ABIDE dataset)	8 brain functional networks from group-ICA	Dual regression	3D CNN classifier^*a*^	77.74%
Reiter et al. (2021)	306 ASD 350 TC (400 participants for each sample)	rs-fMRI data (the ABIDE dataset and data sample from SDSU)	FC between 237 ROIs (the Gordon atlas the HO atlas)	Conditional random forest	Random Forest	62.5% 65% 70% 73.75%

*^a^Machine learning methods that are deep learning methods.*

*Abbreviations: ASD, autism spectrum disorder; fMRI, functional magnetic resonance imaging; rs-fMRI, resting state fMRI; TC, typical control; FC, functional connectivity; ROI, region of interest; AAL, Automated Anatomical Labeling; ABIDE, Autism Brain Imaging Data Exchange; ToM, Theory of Mind; SVM, support vector machine; FA, factor analysis; GNB, Gaussian naïve Bayes; PCA, principal component analysis; RFE, recursive feature elimination; MRMR, maximal relevance and minimal redundancy; UCLA, University of California at Los Angeles; PSO, particle swarm optimization; HOG, histogram of oriented gradients; ICA, independent component analysis; MSDL, multi-subject dictionary learning; L1-SCCA, the L1-norm regularized sparse canonical correlation analysis; SAEs, sparse auto-encoders; SLR, Structured Logistic Regression; LSTM, long short-term memory; DNN, deep neural network; DBN, deep belief network; RBF-SVM, radial basis function-support vector machine; GM, gray matter; WM, white matter; LASSO, least absolute shrinkage and selector operation; SSAE, a stacked sparse auto-encoder; SIMLR, Single-cell Interpretation via Multi-kernel LeaRning; PSD, Power spectral densities; DTL-NN, deep transfer learning neural network; AE, autoencoders; HO, Harvard Oxford; DANN, deep attention neural network; CNN, convolutional neural network; Lo-D-FCs, low-order dynamic functional connectivity networks; Ho-D-FCs, high-order dynamic functional connectivity networks; MTFS-EM, an improved multi-task feature selection method integrating elastic net and manifold regularization; SDSU, San Diego State University.*

## Feature Construction from Raw fMRI Data

Sundry features constructed from raw fMRI data reflect special meanings of brain information. They are important inputs of machine learning and can influence the performance of classification together with the explanation of contributed brain areas to some extent. Therefore, proper feature construction from raw fMRI data of ASD patients and the TCs becomes a crucial step of classification. FC features are most popularly adopted features given that they can reflect particular significance of ASD. Besides, some other meaningful features are also introduced.

### Non-task Static Functional Connectivity Features

Resting-state FC has been proven to be a critical tool in understanding different disease mechanisms and has great potential to provide biomarkers for disease diagnosis (Price et al., 2014). The typically static FCs are constructed by calculating FC between two regions of interest (ROIs) of the brain. It has been reported that altered patterns of brain FCs were suggested as a key neurobiological correlate of the behavioral characteristics of ASD (Neufeld et al., 2017). Increasingly, it has been accepted that ASD is associated with atypical development of multiple interconnected brain systems rather than isolated brain regions (Minshew and Williams, 2007; Uddin et al., 2013a). Furthermore, Hull et al. reviewed the rs-fMRI literatures over how intrinsic connectivity is altered in the autistic brain, with reports of general over-connectivity, under-connectivity, and/or a combination of both (Hull et al., 2017). The whole brain fMRI is usually parcellated into ROIs defined by different atlases, which are either anatomically defined or functionally defined. The commonly used atlas is the anatomical Automated Anatomical Labeling (AAL) atlas (Murdaugh et al., 2012; Wang et al., 2012; Iidaka, 2015; Guo et al., 2017; Aghdam et al., 2018; Bi et al., 2018; Kazeminejad and Sotero, 2019; Liu J. et al., 2020; Tang et al., 2020; Zhao et al., 2020; Reiter et al., 2021). Aside from the AAL, some other atlases were introduced to construct FCs for ASD classification, such as the Power atlas (Power et al., 2014; Chen et al., 2015; Dodero et al., 2015a; Yin et al., 2021), the Craddock 200 (CC200) atlas (Craddock et al., 2012; Kassraian-Fard et al., 2016; Huang et al., 2020; Kazeminejad and Sotero, 2020; Liu J. et al., 2020), the CC400 atlas (Sherkatghanad et al., 2020), the Harvard Oxford (HO) atlas (Desikan et al., 2006; Fredo et al., 2019), the Desikan–Killiany (DK) atlas (Kong et al., 2018; Soussia and Rekik, 2018), and the sulci-based anatomical atlas (Yahata et al., 2016). In particular, the whole brain FCs between 7,266 ROIs were used as classification features (Anderson et al., 2011; Nielsen et al., 2013; Heinsfeld et al., 2018). In addition, FCs derived from specific brain networks were also attractive. For example, Murdaugh et al. adopted FCs between default mode network (DMN) ROIs as classification features (Murdaugh et al., 2012). Different from the above research, Dodero et al. (2015b) obtained FCs by introducing the University of California at Los Angeles (UCLA) Multimodal Connectivity Database, which is an openly available website for brain network analysis and data sharing (Brown et al., 2012).

### Task-Based Static Functional Connectivity Features

Though static FC features are the most adopted features for ASD classification, they are usually but not absolutely restricted to rs-fMRI data. The underconnectivity theory is based largely on analysis of task-related changes in interregional connectivity during tasks (Masona et al., 2008) that involve language, working memory, mental imagery, executive functions, cognitive control, and social cognition. Given that differentiation between ASD patients and TCs is absolutely confirmed, targeted tasks can expand their fMRI differences relative to rs-fMRI. Therefore, FCs generated from task-based fMRI data also attracted the researchers (Murdaugh et al., 2012; Wang et al., 2012; Deshpande et al., 2013). The Theory of Mind (ToM) hypothesis, proposed by Baron Cohen, has emerged as a highly regarded explanation of autistic behavior (Baron-Cohen, 1988a, b, c; Baron-Cohen et al., 1994; Baron-Cohen, 2004). According to this prior knowledge, Deshpande et al. (2013) acquired fMRI data during a ToM task and obtained FCs between 18 ROIs.

### Dynamic or High-Order Functional Connectivity Features

The aforementioned FCs referred to the conventional static brain FCs, which revealed the intrinsic similarities between a pair of ROIs or specific networks. It was recently accepted that dynamic FCs contained more additional knowledge than static FCs. Dynamic FCs can reveal spatiotemporal network properties not observable in static FCs and may reveal more nuanced transient patterns of atypical FC in ASD (Mash et al., 2019). Even so, there are but not many relative ASD classification studies using dynamic FCs compared to static FCs. Price et al. obtained dynamic FCs based on independent components generated from group-independent component analysis (ICA). In their research, it was demonstrated that using FC features over a wide range of time scales was able to substantially increase ASD classification compared with static FC features (Price et al., 2014), indicating that dynamic FCs are the important supplement of static FC features. A high-order morphological brain network based on Pearson correlation was further proposed by Soussia and Rekik to detect more complex interaction patterns between multiple brain regions (Soussia and Rekik, 2018). Moreover, it was noticed that the identified regions at a high-order level are different from those at a lower order, and this may appear to provide complementary discriminative information for more accurate diagnosis (Soussia and Rekik, 2018). Similarly, multi-level, high-order FC networks were put forward by Zhao et al. to serve as ASD classification features (Zhao et al., 2018), and better classification performance was obtained. Two years later, they fused the features extracted from conventional FCs, low-order dynamic FCs, and high-order dynamic FCs for the ASD classification and achieved the best classification performance than any other type of feature fusion (Zhao et al., 2020). It was indicated that the fusion of different-level FCs can supply complementary relevant information for ASD diagnosis, which was consistent with their previous study.

### Other Applied Classification Features

Through analysis of the relative papers, it was obviously found that the commonly adopted classification features are mainly from statics or dynamic functional networks. Besides, some researchers expanded the range of classification features of ASD through different perspectives. Because reduced attention to social stimuli is one of the defining features of ASD, Odriozola et al. (2016) used two visual oddball tasks to investigate brain systems engaged during attention to social (face) and non-social (scene) stimuli. In their work, multivariate activation patterns in the dorsal part of the anterior insula were chosen as classification features. Chanel et al. (2016) acquired fMRI data from two performed experiments and used the beta maps of each condition estimated at the individual step level as features for the classification. The beta map of each individual is high-dimensional containing 186,217 features. Even the rs-fMRI time series and all voxels within the gray matter (GM) mask were directly chosen as classification features (Dvornek et al., 2017; Rane et al., 2017) with the hypothesis that they will carry more useful information than single, static FC measures. In addition, some researchers applied integrated classification features. For example, volumetry analysis, FC MRI analysis, and graph theory via small-world network analysis were introduced to produce the integrated classification features which contained a total of 22 quantitative local and global imaging features (Zhou et al., 2014). Integrated FCs originated from the AAL atlas, the HO atlas, and the Craddock atlas were implemented in the work of Niu et al. (2020). Moreover, for better classification, some researchers applied their proposed methods to obtain classification features, such as histogram of oriented gradients (HOG) features (Ghiassian et al., 2016) and features by factor analysis (Just et al., 2014). As another example of classification feature fusion, Aghdam et al. (2018) computed means of ROIs respectively for rs-fMRI, GM, and white matter (WM) based on the AAL atlas, and the highest classification accuracy was obtained using the fusion of the three features. Based on FCs of the AAL atlas, Tang et al. (2020) introduced fMRI × ROI connectivity for feature supplements.

## Machine Learning Methods and Classification Results

### Summary About Machine Learning Methods and Classification Accuracy Results

High classification accuracy and confirming the most discriminant features are two main purposes for ASD classification. The most discriminant features resulting from ASD classifications can make a good distinction between the two groups and have the potentiality to serve as disease biomarkers. The higher the classification accuracy, the more creditable the confirmed discriminant features. As a traditional classifier, support vector machine (SVM) has been widely used in the classification of brain disorders including ASD in the last decade. The SVM classifier can be linear and non-linear, which was decided by different kernels, such as linear kernel, polynomial kernel, sigmoid kernel, and Gaussian radial basis function (RBF) kernel. Different kernels were chosen according to different features. Eighteen papers in Table 1 applied SVM classifiers benefiting from their classification power. Bi et al. classified the ASD patients and TCs by 96.15% using SVM with RBF kernel (Bi et al., 2018). Dekhil et al. (2018) obtained 91% classification accuracy using the same classification method. Deshpande et al. (2013), Chen et al. (2015), Chanel et al. (2016), Odriozola et al. (2016), Bernas et al. (2018), and Zhao et al. (2018, 2020) all made use of SVM classifiers to discriminate ASD patients and TCs with higher accuracies, which are 92.3% (peak), 85%, 81%, 90.8%, 95.9%, and 86.7%, respectively. However, the choice of SVM classifiers is not the only determining factor for high accuracies, which will be discussed in the next section. Kassraian-Fard et al. (2016) and Liu J. et al. (2020) also applied the SVM classifier but resulted in a lower accuracy probably due to the large multi-site fMRI dataset.

Besides the SVM classifier, some other traditional classifiers are also used in the discrimination of ASD patients and TCs, such as logistic regression (Murdaugh et al., 2012; Wang et al., 2012; Uddin et al., 2013a), random forest (RF) (Chen et al., 2015; Fredo et al., 2019; Reiter et al., 2021), manifold Laplacian (Dodero et al., 2015a), Gaussian naïve Bayes (GNB) (Just et al., 2014), and so on. In order to contrast the effect of different machine learning methods, the scikit-learn library, which contains multiple machine learning methods, was also applied by some researchers for ASD classification (Plitt et al., 2015; Abraham et al., 2017; Rane et al., 2017). In addition, many researchers tried their best to develop novel classification approaches for better performance. Ghiassian et al. proposed (f)MRI HOG-feature-based patient classification (MHPC) learning algorithm to distinguish ASD individuals and TCs by 65% (Ghiassian et al., 2016). Yahataet al. developed a novel machine learning algorithm called Structured Logistic Regression (SLR) classifier and separated the two groups with the accuracy of 85% (Yahata et al., 2016). In addition, Niu et al. employed a proposed multichannel domain-adversarial neural network (DANN) model and further compared it with the existing machine learning methods such as random RF, SVM models, and multichannel deep neural network (DNN), resulting in the best performance of the self-proposed method with an accuracy of 73.2% (Niu et al., 2020).

**FIGURE 2:**
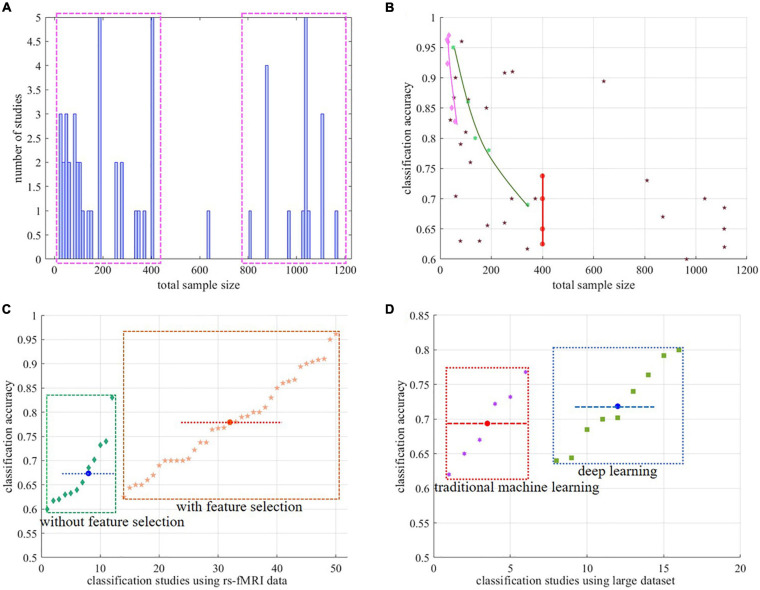
Summary of the involved literatures **(A)** relationship between number of studies and total sample size. **(B)** relationship between classification accuracy and total sample size. The pink diamonds and the others represent the task-based fMRI literatures and the rs-fMRI literatures, respectively. The red dots denote the same classification results by Reiter et al. (2021) and Fredo et al. (2019). The green hexagons denote the classification results of Kazeminejad and Sotero (2020). **(C)** Classification accuracy comparison of studies with feature selection and not, regarding the rs-fMRI dataset. **(D)** Classification accuracy comparison of studies with deep learning and not, regarding the rs-fMRI dataset with more than 800 participants. fMRI, functional magnetic resonance imaging; rs-fMRI, resting state fMRI.

Moreover, as an advanced and popular machine learning method, deep learning was widely applied in the classification of ASD especially since 2017. There are several commonly used models of deep learning, such as, autoencoders (AE), long short-term memory (LSTM), recurrent neural network (RNN), DNN, deep belief network (DBN), and convolutional neural network (CNN) (Du et al., 2018). Fourteen of 47 involved papers applied deep learning methods for the classification as detailed in Table 1. Purely from the perspective of classification accuracy, the performance of advanced deep learning methods is comparable with that of traditional ones. In fact, classification accuracy was affected by not only machine learning methods but the dataset, constructed features, and feature selection methods, which will be discussed in detail in section “Factors on Classification Accuracy.” Therefore, the scientific and meaningful comparison on performance of deep learning and traditional machine learning should be carried out under the same condition. Classification accuracies were compared between deep learning and traditional machine learning in Figure 2D using papers with more than 800 participants from the ABIDE dataset. Generally speaking, deep learning outperformed traditional machine learning. In the work of Thomas et al. (2020) CNN was reported to achieve comparable results with SVM as shown in Table 2. Apart from this, eight other papers with deep learning reported their better performance than traditional machine learning methods under the same condition detailed in Table 2 (Heinsfeld et al., 2018; Kong et al., 2018; Li et al., 2018; Huang et al., 2020; Kazeminejad and Sotero, 2020; Sherkatghanad et al., 2020; Thomas et al., 2020; Yang et al., 2021; Yin et al., 2021). It is reasonable to believe that deep learning holds better classification power than the traditional ones.

**TABLE 2:** Accuracy comparison between deep learning and othermachine learning.

Study	Deep learning accuracy	Othermachine learning accuracy
Dvornek et al. (2017)	LSTM classifier: 68.5%	SVM classifier: 66.9% MHPC classifier: 59.2%
Heinsfeld et al. (2018)	DNN classifier: 70%	SVM classifier: 65% RF classifier: 63%
Kong et al. (2018)	DNN classifier: 90.39%	RF classifier:74.46%
Sherkatghanad et al. (2020)	CNN classifier: 70.22%	SVM classifier: KNN classifier: RF classifier:
Kazeminejad and Sotero (2020)	A multilayer perceptron: 64.4% (average)	LR classifier: 61% (peak) RF Classifier: 63% (peak) RBF-SVM classifier: 67% (peak)
Thomas et al. (2020)	CNN classifier: 64%	SVM classifier: 66% (comparable)
Huang et al. (2020)	DBN classifier: 76.4%	DNN classifier: 70% LSTM classifier: 68.5% L-SVM classifier: 68.5% SVC classifier: 66.9% LOOCV classifier: 60% RBF-SVM: 59.2%
Yin et al. (2021)	DNN classifier: 79.2%	L-SVM classifier: 68% Medium-SVM classifier: 72.2% Coarse Gaussian-SVM classifier: 66.9% Medium-KNN classifier: 72.4% Cosine-KNN classifier: 72.6% Weighted KNN classifier: 72.4%
Yang et al. (2021)	CNN classifier: 77.74%	AE-MLP classifier: 68.56% SVM classifier: 62.97% RF classifier: 60.62% Conv GRU-CNN classifier:67%

*Abbreviations: LSTM, long short-term memory; SVM, support vector machine; DNN, deep neural network; RF, random forest; KNN, K-nearest neighbor; LR, logistic regression; RBF-SVM, radial basis function-support vector machine; L-SVM, linear support vector machine; SVC, support vector classification; LOOCV, leave-one-out cross-validation; AE-MLP, autoencoders-multilayer perceptron; CNN, convolutional neural network; GRU, gated recurrent unit.*

### Factors on Classification Accuracy

High accuracy is a critical goal of classification between ASD individuals and TCs aiming to determine biomarkers for ASD. Through analysis of the involved literatures, several factors that substantially impacted classification accuracy were summarized.

#### Task-Based fMRI Dataset

Many studies have proved the brain difference between ASD individuals and TCs. There is a hypothesis that the degree of alteration in the representation of *self* in individuals with autism would be related to behavioral measures of various social abilities, such as thinking, face processing, and ToM (Just et al., 2014). Thus, fMRI data with correlated tasks are commonly used for ASD classification studies (Murdaugh et al., 2012; Wang et al., 2012; Deshpande et al., 2013; Just et al., 2014; Chanel et al., 2016; Odriozola et al., 2016). It was found that classifications using task-based fMRI data usually obtained high accuracies as detailed in Table 1 and represented by the pink diamonds in Figure 2B, ranging from 82.8% to 97%. Compared to the rs-fMRI data used in the classification, the task-based fMRI data are an important factor for high classification accuracies. But for task-based fMRI data, it is difficult to acquire large datasets. Most task-based fMRI studies relatively involved small samples of usually less than 50 subjects (Poldrack and Gorgolewski, 2015). Interestingly, we found that the less samples, the higher the classification accuracy as shown in Figure 2B. It is worthy of note that the classification accuracy may decrease with an increase in the number of individuals in the task-based fMRI studies, which necessitates further demonstration.

#### Contribution of Feature Selection

Besides the factor of the task-based fMRI data, another important factor for high classification accuracy is feature selection. Feature selection methods play an important role in classifications because of the high-dimension property of fMRI data even after relative features have been constructed. Proper feature selection can further reduce the dimensionality of features, enhance classification accuracy, facilitate visualization of the data, and lead to faster classification (Guyon and Elisseeff, 2003; Kassraian-Fard et al., 2016). To realize the importance of feature selection in classification, it gradually became an indispensable part of ASD classification studies. Nevertheless, 16 of the 47 papers in this review did not introduce separate feature selection methods as detailed in Table 1, and high dimensional features were directly applied as the inputs of classifiers (Nielsen et al., 2013; Heinsfeld et al., 2018) resulting in lower classification accuracies. The performance of ASD classification with and without feature selection methods was also compared in some works. Chen et al. (2015) achieved 90.8% classification accuracy using the top 100 features with the highest variable importance compared with 58% accuracy without feature selection. Guo et al. (2017) demonstrated that classification with feature selection outperformed that without feature selection method by 9.09%, and different feature selection methods could bring different classification results. Worth mentioning was that the ASD classification studies using feature selection method in the review can averagely bring better classification accuracies than those without it as statistically illustrated in Figure 2C. In summary, feature selection methods made an indelible contribution to the performance of ASD classification.

#### The Property of the Dataset

Another non-ignored factor of the high classification accuracy is the property of the dataset applied for classification such as sample size and data heterogeneity. The total sample size of the involved literatures mostly concentrated less than 400 or more than 800 as shown in Figure 2A. The relationship between classification accuracy and sample size was illustrated in Figure 2B, in which the pink diamonds and others represent the results of the literatures involving tasks or not, respectively. Classification across multiple sites has to accommodate additional sources of variance in subjects, scanning procedures, and equipment in comparison to single-site datasets (Nielsen et al., 2013) and usually results in low classification accuracies detailed in Table 1. However, it is worth noting that several works in Table 1 applying the fMRI data from the ABIDE dataset brought high accuracies from 81% to 96.15% (Price et al., 2014; Chen et al., 2015; Bi et al., 2018; Kong et al., 2018; Zhao et al., 2018, 2020; Kazeminejad and Sotero, 2019). Through analysis, the commonality of datasets applied in these papers is that they are all subsets of the large dataset according to some special selection criteria, such as site limitation, single protocol, or age-related selection, which can alleviate data heterogeneity to some extent and improve the classification accuracy. Eslami et al. (2019) evaluated their proposed machine learning method on all data and each site data of the ABIDE dataset respectively, resulting in 70.1% for all data and 80% peak accuracy for the Oregon Health & Science University (OHSU) site. Likewise, several works that focused on childhood and adolescent fMRI data also attained high accuracies from 83% to 91% (Uddin et al., 2013a; Price et al., 2014; Iidaka, 2015; Bernas et al., 2018; Dekhil et al., 2018) following the principal of the above age-related selection. In the work of Kazeminejad et al., the dataset including 817 participants was split into five age ranges, and the best classification accuracies for each range were obtained ranging from 69% to 95% (Kazeminejad and Sotero, 2019). In accordance with the task-based fMRI studies, an approximately linear relationship between classification accuracies and sample size was discovered in their work as illustrated in Figure 2B. In addition, the same sample size could result in different classification accuracies due to different heterogeneity of the dataset, which was illustrated by the red dots in Figure 2B (Fredo et al., 2019; Reiter et al., 2021). All in all, the property of the applied dataset can definitely influence the classification result to some extent.

#### The Choice of Atlas

**FIGURE 3:**
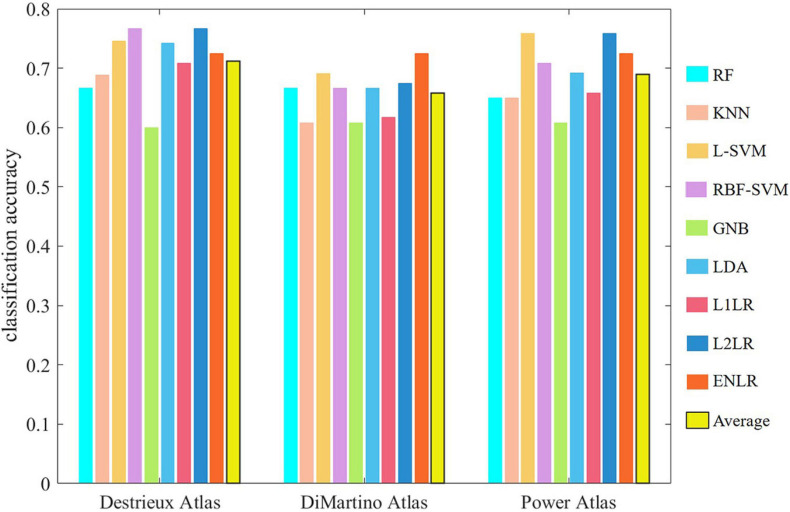
Classification accuracy comparison based on different choice of atlas in the work of Plitt et al. (2015). RF, random forests; KNN, K-nearest neighbor; L-SVM, linear support vector machine; RBF-SVM, Gaussian kernel support vector machine; GNB, Gaussian naïve Bayes; LDA, linear discriminant analysis; L1LR, L1 logistic regression; L2LR, L2 logistic regression; ENLR, elastic-net logistic regression; Average, average of the above nine methods.

As discussed in section “FEATURE CONSTRUCTION FROM RAW FMRI DATA,” atlases of anatomical, functional parcellation, and data-driven extraction were applied for FC feature construction in ASD classification. It was believed that the choice of atlas could influence classification accuracy to some extent (Plitt et al., 2015; Abraham et al., 2017; Dadi et al., 2019; Liu Y. et al., 2020; Yin et al., 2021). Plitt et al. addressed the impact of three different brain atlases on classifications (Power et al., 2011; Plitt et al., 2015), which are the DiMartino atlas, the Destrieux atlas, and the Power atlas. The Destrieux atlas slightly outperformed the other two as illustrated in Figure 3, indicating that different anatomical atlases influenced the classification accuracy indeed. Meanwhile, Abraham et al. considered three different predefined atlases and four data-driven atlases and compared their classification performance (Abraham et al., 2017). The three predefined atlases are the anatomical HO atlas (Desikan et al., 2006), the functional Yeo atlas (Yeo et al., 2011), and the functional Craddock atlas (Lashkari et al., 2009), while the four data-driven atlases were derived based on K-means, Ward’s clustering, ICA, and multi-subject dictionary learning (MSDL). Data-driven atlases were reported to perform more poorly than the predefined atlases except the MSDL-based atlas, and the functional HO atlas led to maximal performance. In the work of Dadi et al., functional atlases were reported to lead to better prediction than anatomical atlases, and the MSDL-based atlas was found to perform comparably (Dadi et al., 2019). The MSDL-based atlas exhibited robust performance out of all the anatomical and data-driven atlas approaches (Yin et al., 2021). In a summary, the choice of atlas played an important role on the prediction accuracy. Apart from this, the great meaningful contribution of the choice of atlas was the explanation of discriminant features or biomarkers derived from classification.

#### Development of Machine Learning Method

As the core of classification, machine learning methods are undoubtedly a crucial factor on classification accuracies. Different machine learning methods and their variants were introduced to fMRI data classification study for better classification results, determination of discriminant features, ASD biomarkers exploration, or just method innovation. Among traditional shallow machine learning methods, SVM was recognized as a powerful one than others. With the appearance and rapid development of deep learning, it was widely applied in ASD classification. Apart from its lack of interpretability restrictions and being time consuming, deep learning has shown great potential power in classification compared with traditional machine learning methods as discussed in section “Summary About Machine Learning Methods and Classification Accuracy Results.” Further and deeper study on deep learning can continue to promote the development of ASD classification. The most promising focus on the application of machine learning to the neuroimaging field may be to create specific methods for the special properties of fMRI.

## Significant Results Summarized from Involved Literatures

It is well known that one major goal of the classifications between ASD and TC is to obtain high accuracies, while another is to determine the informative brain regions or networks contributing to the classifications with the ultimate goal of yielding a possible biomarker of ASD. The higher the accuracy, the more trustworthy the identified brain regions or networks. Not only the informative brain areas or networks were determined, but the physiology and behavior explanation about them were exhibited in most of the papers.

### Consistent Results From FC Classification Using the AAL Atlas

Given that FCs were chosen by many researchers as classification features, we summarized informative brain regions and networks determined by classifications between ASD individuals and TCs using FCs as features in Table 3. The brain regions and networks are more comparable in different literatures which applied the same brain atlas regardless of different pattern classification methods. As an anatomically defined atlas, the AAL atlas was frequently adopted in ASD classifications (Murdaugh et al., 2012; Wang et al., 2012; Iidaka, 2015; Guo et al., 2017; Bi et al., 2018; Liu J. et al., 2020; Tang et al., 2020; Zhao et al., 2020). The informative brain regions and networks were determined separately from FC classifications as shown in Table 3 except the work of Guo et al. (2017) and Tang et al. (2020). The common brain regions determined by classifications of the several above literatures included the right posterior cingulate cortex (PCC), the left PCC, and the right thalamus. The conclusion derived from the work of Wang et al. (2012, 2017, 2020) is that weak functional connections between the frontal lobe and the rest of the cortex occurred in ASD patients compared with the TCs. Dodero et al. (2015a) obtained eight informative connections through FCs classification detailed in Table 3, in which red connections identify higher connectivity in healthy subjects while blue connections identify a higher connectivity strength in autistic subjects. Guo et al. (2017) found 32 most significant FCs between the two groups mainly from or across different pre-defined brain networks including the default mode, cingulo-opercular, frontal-parietal, and cerebellum. The detailed significant brain areas about the above papers are shown in Table 3.

**TABLE 3:** Summary of informative brain regions or networks determined by classification between ASD and TD usingFCs as features.

Authors	Brain atlas	Informative brain regions or networks contributing to theclassification between ASD and TD
Anderson et al. (2011)	7,266 regions	The DMN, superior parietal lobule, fusiform gyrus and anterior insula
Wang et al. (2012)	The AAL atlas	Weak functional connections between the frontal lobe and the rest of the cortex
Murdaugh et al. (2012)	The AAL atlas	The PCC, PCUN, and MFC
Nielsen et al. (2013)	7,266 regions	The DMN, parahippocampaland fusiform gyri, insula, Wernicke area, and intraparietal sulcus
Deshpande et al. (2013)	18 self-defined ROIs	Functional connections between the fusiform face area and middle temporal gyrus
Iidaka (2015)	The AAL atlas	The medial part of the superior frontal gyrus, anterior and posterior cingulate cortices, and thalamus
Chen et al. (2015)	The Power atlas	Default mode, somatosensory/motor (hand region), and visual networks The left anterior cingulate gyrus, bilateral postcentral gyrus, right PCUN, left calcarine sulcus, the left paracentral lobule and the right postcentral gyrus
Plitt et al. (2015)	The Power atlas The Destrieux atlas The DiMartino atlas	The insula, ventromedial prefrontal cortex, anterior, middle, and posterior regions of cingulate cortex, supplementary motor cortex, anterior temporal lobes, posterior aspects of the fusiform gyrus, posterior superior temporal sulcus, temporal parietal junction, intraparietal sulcus, and inferior and middle frontal gyri, bilaterally Default-mode network, the frontal-parietal control network
Yahata et al. (2016)	Sulci-based anatomical atlas	16 discriminative functional connections The cingulo-opercular network
Dodero et al. (2015b)	The Power atlas	Red: left precentral gyrus-left occipital pole, left precentral gyrus-left precentral gyrus, left superior frontal gyrus-right lateral occipital cortex, right superior frontal gyrus-right parietal operculum Blue: right frontal medial cortex-right precentral gyrus, left caudate-right precentral gyrus, left putamen-right precentral gyrus, right frontal pole-right lateral occipital cortex
Abraham et al. (2017)	The HO atlas The Yeo atlas The CC200 atlas	DMN, Pareto-insular network and semantic ROIs
Guo et al. (2017)	The AAL atlas	32 most significant FC mainly from or cross different pre-defined brain networks including the default-mode, cingulo-opercular, frontal-parietal, and cerebellum
Heinsfeld et al. (2018)	7,266 regions	The regions with the highest anticorrelation: paracingulate gyrus, supramarginal gyrus, and middle temporal gyrus The regions with the highest correlation: occipital pole, and lateral occipital cortex; superior division
Bi et al. (2018)	The AAL atlas	The right IFG (opercular part), the right PCUN, superior frontal gyrus (orbital part), the left inferior occipital gyrus, the right hippocampus, the bilateral superior frontal gyrus (dorsolateral), the right median cingulate and paracingulate gyri, the right posterior cingulate gyrus, the left supramarginal gyrus, the right thalamus, the right superior, and middle temporal gyrus
Bhaumik et al. (2018)	The Brodmann’s areas ROIs	Dorsolateral prefrontal cortex, somatosensory association cortex, primary auditory cortex, inferior temporal gyrus and temporopolar area
Fredo et al. (2019)	The Gordon’s cortical atlas The HO atlas	COTC, visual, DA, DMN, and SMH
Liu Y. et al. (2020)	The CC200 atlas	Lower correlation between the anterior and posterior DMN in autistic individuals
Sherkatghanad et al. (2020)	The CC400 atlas	The right supramarginal gyrus, the fusiform gyrus, the cerebellar vermis (C115, C188, C247, and C326)
Zhao et al. (2020)	The AAL atlas	Precentral gyrus, middle frontal gyrus, middle cingulate gyrus, posterior cingulate gyrus, amygdala, angular gyrus
Huang et al. (2020)	The CC200 atlas	20 discriminative functional connections
Reiter et al. (2021)	The Gordon atlas The HO atlas	COTC, visual, SMH, DMN, and DA

*Abbreviations: ASD, autism spectrum disorder; TD, typically developing; FC, functional connectivity; DMN, default mode network; AAL, Automated Anatomical Labeling; PCC, posterior cingulate cortex; PCUN, precuneus; MFC, medial prefrontal cortex; ROI, region of interest; HO, Harvard Oxford; IFG, inferior frontal gyrus; COTC, cingulo-opercular task control; DA, dorsal attention; SMH, somatosensory motor hand.*

### Consistent Results About the Default Mode Network

The DMN comprised several dispersed cortical nodes including the anterior cingulate/medial prefrontal cortices and the PCC (Raichle et al., 2001; Greicius et al., 2003; Buckner et al., 2008; Washington et al., 2014). Functionally, the DMN is considered to relate to self-referential cognition including domains of known impairment in ASD (Chen et al., 2015), which have been reported in some previous non-classification research (Monk et al., 2009; Assaf et al., 2010; Minshew and Keller, 2010; Zielinski et al., 2012; Martino et al., 2013; Redcay et al., 2013). Notably, some common regions determined by the aforementioned literatures using the AAL atlas belong to the DMN. Early in 2011, Anderson et al. have reported the phenotypic pattern of impaired communication within and between the DMN and attention control networks in ASD through classification between the two groups (Anderson et al., 2011). Soon afterward, a series of ASD classification studies were carried out and resulted in consistent conclusions about the DMN. As depicted in Table 3, impaired FCs related to the DMN were obtained in several literatures (Nielsen et al., 2013; Chen et al., 2015; Yahata et al., 2016; Abraham et al., 2017; Heinsfeld et al., 2018; Fredo et al., 2019; Reiter et al., 2021). Besides, Murdaugh et al. (2012) demonstrated that deactivation and connectivity of the DMN were altered in individuals with ASD at a high classification accuracy of 96.3%. The impaired DMN was also reported in the research by Plitt et al. together with the frontal-parietal control network (Plitt et al., 2015). In addition, seven distinctive fronto-parietal and temporal networks between ASD patients and TCs were reported in the work of Bernas et al. (2018) one of which was DMN. Lower correlation was proved between the anterior and posterior DMN in autistic individuals (Liu Y. et al., 2020). Both dorsal DMN (dDMN) and precuneus (PCUN) achieved better accuracies than other brain networks in the work of Yang et al. (2021), which are the subnetworks of the DMN. Taken together, the DMN is undoubtedly a crucial component of the underlying neurobiology and has the potentiality to serve as a biomarker of ASD.

### Partial Hemispheric Distribution of Discriminant FCs or Brain Regions

It has been reported that the distributed patterns of functional abnormalities are over the whole brain of ASD patients (Zhao et al., 2018). Actually, in the work of Nielsen et al. (2013), a homogenous regional distribution of connectivity abnormalities in autism was argued against and replaced by a heterogeneous spatial distribution of connectivity disturbances that involves specific brain regions. The conclusion of partial hemispheric distribution of informative brain regions and networks in autism were identically obtained in several other involved literatures. There were significantly more regions in the right hemisphere than in the left among the brain regions involved in the 16 FCs identified in the study of Yahata et al. (2016) with the left intra-hemispheric FCs absent. Likewise, significant discriminative connections between the two groups were mostly located in the right hemisphere, and there were more involved brain areas in the right hemisphere than in the left, which was detailed in the work of Dodero et al. (2015a). Odriozola et al. (2016) also found that children with ASD displayed greater activation of the right insula when viewing deviant faces vs. scenes in contrast to their TCs (Odriozola et al., 2016). In addition, the best performance was achieved in distinguishing between ASD/TC subjects for the right hemisphere by Soussia and Rekik indicating that the right hemisphere features may have more discriminative power (Soussia and Rekik, 2018). Though not all relative papers involved the conclusion about partial hemispheric distribution, it undoubtedly supplied a novel understanding and a new prospective for ASD.

### Identification of the Potential ASD Biomarker

As a prime conception in the field of psychiatric neuroimaging research, *biomarkers* have successfully attracted enough attraction of researchers especially with the emergence and development of machine learning. More than 10 years ago, machine learning methods were thought to be a promising method to reveal brain states that discriminate patients from controls and thus constitute a valuable tool to identify potential biomarkers (Mourão-Miranda et al., 2005; Pereira et al., 2009). Notably, 38 of 47 papers in the review mentioned “*biomarker*” to some extent, which appropriately proved the collaborative efforts from various research teams to explain or identify the objective biomarker of ASD using machine learning methods and fMRI data. Plitt et al. (2015) indicated that FC classification of autism identifies highly predictive brain features but falls short of biomarker standards for several reasons, such as establishing standard analytic techniques, demonstrating biomarkers robustness to variability across larger numbers of individuals and sites, and addressing the diagnostic potential of brain-based biomarkers. It was thought that the predicted autistic neural patterns determined by classification are anticipated to serve as reproducible biomarkers and important in early diagnosis and treatment (Kong et al., 2018; Huang et al., 2020). Resting-state FC measures were proved to be potential diagnostic biomarkers for ASD in several studies (Deshpande et al., 2013; Price et al., 2014; Plitt et al., 2015; Bhaumik et al., 2018; Kong et al., 2018; Soussia and Rekik, 2018; Fredo et al., 2019). The study of Iidaka (2015) indicated that an intrinsic connectivity matrix constructed from rs-fMRI data could yield a possible biomarker of ASD restricted to children and adolescents. It was proposed that the high-order FC could be affected in ASD compared with the traditional FC and thus can be used as effective biomarkers for ASD diagnosis in the work of Zhao et al. (2018). In the study of Bernas et al. a change in the coherence of temporal neurodynamics is identified to be a biomarker of ASD (Bernas et al., 2018). Thomas et al. (2020) reported that hidden somewhere in the high-dimensional spatio-temporal signal are the biomarkers that could distinguish between healthy and psychiatric subjects. A strong negative correlation between the left precuneous cortex and the left superior frontal gyrus was anticipated to serve as reproducible biomarkers (Huang et al., 2020). In the work of Yang et al. (2021), the dDMN, PCUN, and salience network (SN) are suggested to be highly different between ASD and normal control (NC) and have the potential to be reliable biomarkers for the identification of ASD. Though most works of relative papers failed to identify definite and replicated biomarkers of ASD, efforts from different research teams have put the identification of the ASD biomarker forward, and it was believed that an ideal biomarker would be derived through the continuous work.

### Some Other Specific Meaningful Results

In the work of Heinsfeld et al. (2018), the most contributing conclusion is the anterior-posterior disruption in the connectivity of ASD, which has been reported to be shown in previous task-related (Adam et al., 2004; Kana et al., 2009) and rs-fMRI studies of ASD patients (Cherkassky et al., 2006). Similarly, the right supramarginal gyrus, the fusiform gyrus, and the cerebellar vermis were found to play a significant role in the diagnosis of autism (Sherkatghanad et al., 2020), which was another evidence about the disruption of anterior-posterior brain connectivity in ASD. Lower correlation between the anterior and posterior DMN was demonstrated in autistic individuals than controls (Liu Y. et al., 2020). Brain regions related to social communication, emotion expression, language comprehension, and action coordination were determined, such as inferior frontal gyrus, amygdala, angular gyrus, and hippocampus (Zhao et al., 2018). In the work of Uddin et al. the SN was identified to play a critical role in discriminating children with ASD from TC and could be a hallmark of ASD (Uddin et al., 2013b) with the explanation that regions within the SN are implicated in multiple functions, ranging from attention to interception and subjective awareness (Craig, 2011). Changes in caudate volume, caudate-cortical FC, and inferior frontal gyrus FC were reported to be highly informative in the classification of the two groups (Zhou et al., 2014). Moreover, the social brain has been accepted to be impaired in individuals with ASD, which was further confirmed from the perspective of fMRI classification through four literatures (Deshpande et al., 2013; Just et al., 2014; Chanel et al., 2016; Bernas et al., 2018). Though most involved literatures identified contributing brain regions or networks, some still gave more prominence to the methods and results of classifications and ignored the determination of informative brain regions and networks (Kassraian-Fard et al., 2016; Dvornek et al., 2017; Ktena et al., 2017; Kong et al., 2018; Kazeminejad and Sotero, 2019; Liu J. et al., 2020; Niu et al., 2020; Yin et al., 2021).

## Future Work Direction

In this paper, we summarized 47 literatures involved in fMRI data classification between ASD individuals and TCs. Most researchers expected to derive the biomarkers of ASD through classification studies and have made some progress in deed, but the overall assessment of classification of ASD using fMRI data thus far falls short of biomarker standards. Despite this, several work directions may need to be paid more attention by researchers: (1) Considering the factors for high classification accuracies, development of novel feature selection methods should be an important work direction for classifications between ASD individuals and TCs, which could facilitate machine learning methods to determine the most discriminant features. (2) Another pivotal work direction is to obtain advanced machine learning methods by improving the existing methods and combining the superiority of different methods, especially trying to develop specific machine learning methods for the special properties of fMRI. (3) Non-invasive rs-fMRI data classification studies between ASD individuals and TCs will still be the prominent work direction especially since the release of the ABIDE dataset. More robust and replicated biomarkers are more likely derived from big datasets. (4) Age is an important factor in the ASD diagnosis. Age-specific research can significantly reduce the heterogeneity of ASD dataset and increase the classification rate. Therefore, the age-specific research of ASD will be a valuable research direction. (5) Because of the higher classification accuracies for task-based fMRI data, it may be a new work direction to design some easily operated and efficient tasks to acquire fMRI data to form large datasets for further research. In the future, developed feature selection and machine learning methods combined with large rs-fMRI datasets or easily operated task-based fMRI dataset may appear to serve as a promising diagnostic tool for ASD.

## Author Contributions

All authors listed have made a substantial, direct and intellectual contribution to the work, and approved it for publication.

## Conflict of Interest

The authors declare that the research was conducted in the absence of any commercial or financial relationships that could be construed as a potential conflict of interest.

## Publisher’s Note

All claims expressed in this article are solely those of the authors and do not necessarily represent those of their affiliated organizations, or those of the publisher, the editors and the reviewers. Any product that may be evaluated in this article, or claim that may be made by its manufacturer, is not guaranteed or endorsed by the publisher.

## Footnotes

1
http://fcon_1000.projects.nitrc.org/indi/abide/
